# Evans Syndrome: A Case Report

**DOI:** 10.31729/jnma.7421

**Published:** 2022-05-31

**Authors:** Sanam Dhakal, Sulochana Neupane, Ankit Mandal, Surya Bahadur Parajuli, Sulav Sapkota

**Affiliations:** 1 Birat Medical College and Teaching Hospital, Biratnagar, Morang, Nepal; 2 Department of Community Medicine, Birat Medical College and Teaching Hospital, Biratnagar, Morang, Nepal; 3 Department of Medical Hematology and Oncology, Birat Medical College and Teaching Hospital, Biratnagar, Morang, Nepal

**Keywords:** *case reports*, *immunoglobulins*, *neutropenia*, *thrombocytopenia*

## Abstract

Evans syndrome is defined as the concomitant or sequential association of warm autoimmune hemolytic anaemia with immune thrombocytopenia, and less frequently autoimmune neutropenia. It is associated with non-cross-reacting auto-antibodies directed against antigens specific to red blood cells, platelets or neutrophils. Clinical symptoms could be related to hemolysis and thrombocytopenia. Evans syndrome is a rare diagnosis of exclusion. The first-line treatment of Evans syndrome is intravenous corticosteroids or intravenous immunoglobulins and second-line treatment with rituximab or splenectomy for those who are refractory to steroids. Here is a case of a 50-year-old-female who presented with bleeding from the mouth and gums, bluish patches over the shin and trunk along with generalised weakness and severe backache. We are interested in reporting this case because the presentation of patients with such scenarios on our part will compel the treating physician to overlook Evans syndrome and get it underdiagnosed.

## INTRODUCTION

Evans Syndrome is defined as the concomitant or sequential association of warm Autoimmune Hemolytic Anaemia (AIHA) with Immune Thrombocytopenia (ITP), and less frequently autoimmune neutropenia.^1,2^ In this rare disorder body makes antibodies against one's self Red Blood Cells (RBCs), White Blood Cells (WBCs) and platelets. Individuals may manifest signs and symptoms due to anaemia, low platelets and leukocytes. When AIHA and ITP occurred concomitantly, the diagnosis must exclude differentials such as thrombotic microangiopathies, anaemia due to bleeding complicating ITP, vitamin deficiencies, myelodysplastic syndromes, paroxysmal nocturnal hemoglobinuria, or specific conditions like Hemolysis, Elevated Liver enzymes and Low Platelets (HELLP) when occurring during pregnancy.^2^

## CASE REPORT

A 50-year-old-female referred from Makalu hospital, Biratnagar was admitted to the Intensive Care Unit (ICU) of Birat Medical College and Teaching Hospital with the complaints of bleeding from the mouth and gums, bluish patches over the shin and trunk along with generalised weakness and severe backache for 7 days. She also complained of passing stool mixed with blood. She had no history of fever, shortness of breath, cough, or headache. Blood was not transfused till then. She was non-diabetic, normotensive and euthyroid. She used to smoke cigarettes occasionally but denies alcohol and unnecessary drug intake.

At presentation, she was ill-looking, conscious, cooperative and well oriented to time, place and person with the Glasgow Coma Scale (GCS) 15/15. No other significant findings on general and systemic examination. Her vitals were stable.

All serological, biochemical and microbiological tests were done which were normal. Her haemoglobin, RBC count and platelet were significantly low with neutrophilic leukocytosis. Peripheral blood smear report showed anisopoikilocytosis with the presence of microcytes, four nucleated RBC/ 100 WBCs, neutrophilic leukocytosis and reduced platelet with no other atypical cells and hemoparasites. The direct Coombs test was positive.

The main differentials of Evans syndrome are hemolytic uremic syndrome and disseminated intravascular coagulation. To differentiate Evans syndrome from these conditions a detailed clinical history centred on determining the risk factors such as infections, malignancies, autoimmune diseases, drugs or a family history of immune disorders complemented with a thorough physical examination focused on signs of anaemia or thrombocytopenia is needed. It is important to mention that primary ES is a diagnosis of exclusion that is confirmed by a combination of tests i.e., complete blood count showing low haemoglobin and platelets, peripheral blood smear with reticulocytosis and finally the direct Coombs test being positive.

For her initial symptoms, at the time of admission 1 pint of packed blood cells was transfused and she was monitored giving antibiotics and Vitamin B12. There was a slight improvement in her blood parameters and a plan was made for a peripheral blood smear and direct Coomb's test. She was kept monitored on intravenous steroids for which she was responsive and her condition was gradually improving both clinically and hematologically but hadn't attained the normal range. She was further transfused with 1 pint of packed cell volume on day 3 and 1 pint of platelet-rich plasma each on day 3, day 4 and day 7. Altogether she got 2 pints of blood and 3 pints of PRP transfused. The dose of steroid was tapered and an additional antibody rituximab was started. Her vitals, blood indices and fluid input/output were constantly monitored and were considerable.

The outcome of the above treatment came to be good. Her condition gradually improved and she was discharged from the hospital on the 14^th^ day and visits the doctor weekly. On her first follow up there were no fresh complaints. A full blood count was done for monitoring and she was kept continuous with weekly Rituximab for the first 3 weeks. Then after, weekly steroid pulse therapy was started which was equally responsive. On subsequent follow up her blood count is assessed. It has been her 8^th^ week of follow up and is doing well.

**Figure 1:**
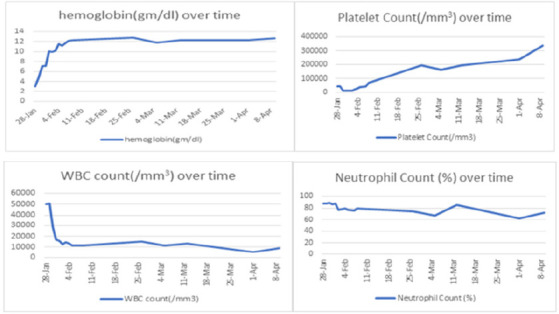
Blood parameters.

Her blood parameters during the hospital stay and follow-up are shown (Figure 1).

## DISCUSSION

The symptoms of Evans syndrome may be similar in nature to leukaemia and lymphoma; these illnesses must be ruled out before a diagnosis is made. With a low RBC count, symptoms may include: jaundice, dark brown urine, pale skin, weakness, fatigue, shortness of breath; with a low platelet count, symptoms may include: increased bruising, petechiae: tiny red dots under the skin that are a result of very small bleeds into the skin, increased bleeding symptoms, such as a bloody nose or heavy menses; with a low neutrophil count, symptoms may include: fevers, mouth sores, increased bacterial infections.^3^ Thirty-two years female presented with a fatal complication of Intracranial haemorrhage in one of the tertiary care centres in Nepal, she was kept on a high dose of intravenous mannitol, platelet-rich plasma, steroids and immunomodulator azathioprine but unfortunately, she died due to noncompliance with therapy.^4^ In our case, the woman presented with petechial patches, gum bleeding, weakness, backache, and blood mixed stool but there were no infections and deadly complications. The symptoms and signs of our cases were similar to those highlighted in other published articles mentioned above. Also, thrombosis as a complication of ITP and AIHA has been well documented in the medical literature; however, there are a limited number of Evans Syndrome which report thrombotic complications.^5^ The association of thrombosis was unfamiliar in our case.

Evans syndrome should be kept in differentials of the cases where lab parameters suggest critically low platelet count with low haemoglobin level. A diagnosis of Evans syndrome is based upon the identification of characteristic symptoms, a detailed patient history, a thorough clinical evaluation, and a variety of specialised tests. No specific test is conclusive for Evans syndrome and a diagnosis is made after excluding other possible diagnoses. Evans syndrome is a diagnosis of exclusion. Specifically, a diagnosis of Evans syndrome may be made when autoimmune hemolytic anaemia (with a positive direct Coombs test) and ITP occur in the same patient even if not at the same time.^6^ Also, the diagnosis in our case was also made based on signs and symptoms and also the direct Coombs test was positive.

Steroids like prednisone and prednisolone are the first-line therapy, with intravenous immunoglobulin administered as a life-saving resource in cases of severe immune thrombocytopenic purpura manifestations. Second-line treatment for refractory Evans syndrome includes rituximab, mofetil mycophenolate, cyclosporine, vincristine, azathioprine, sirolimus and thrombopoietin receptor agonists.^1^ The woman in our case was also managed with blood transfusion, and platelet transfusion and did well with rituximab and corticosteroids and our treatment was parallel with the treatment modalities highlighted in other literature. We started with intravenous steroids and switched to antibody rituximab when she transiently became steroid-refractory and then now, she is well responding to weekly steroid pulse therapy. Steroids act mainly by clearing the macrophages which destroy one's own red cells and platelets. Concentrated immunoglobulin G from human plasma donors blocks the FCy receptor of the macrophages but it remains a controversial therapy. In cases unresponsive to immunosuppressive agents, hematopoietic stem cell transplantation has been successful, although it is necessary to consider its potential serious adverse effects.^1^

Evans syndrome runs a more benign course in pregnancy than in a non-pregnant state (notable neutropenia does not occur) and very often resolves post-delivery.^7^
